# Assessing Personality in Daily Life: Variability Between and Within Persons

**DOI:** 10.1093/geroni/igab046.036

**Published:** 2021-12-17

**Authors:** Giselle Ferguson, Giancarlo Pasquini, Andreas Neubauer, Stacey Scott

**Affiliations:** 1 Stony Brook University, Stony Brook University, New York, United States; 2 Stony Brook University, Stony Brook, New York, United States; 3 Leibniz Institute for Research and Information in Education, Frankfurt, Mecklenburg-Vorpommern, Germany

## Abstract

Trait personality measures may not be able to detect subtle personality changes and fluctuations which may be indicative of cognitive impairment. Measuring personality in daily life may allow sufficient sensitivity to capture this within-person variability. Eighty-six older adults from the Einstein Aging Study completed items assessing daily extraversion and neuroticism for a median of 17 days. Using separate unconditional models, we calculated the proportions of variance in daily extraversion and neuroticism that were due to between-person and within-person variability. Variability in daily extraversion was relatively evenly related to between-person differences and within-person fluctuation (Intra-Class Correlation [ICC] = 0.576), but the majority of variability in daily neuroticism was at the between-person level (ICC = 0.730). Thus, although these daily assessments were sensitive enough to capture within-person variability in personality in daily life, different traits may exhibit more or less of this variability.

